# Association of cumulative early medical events and neurodevelopmental conditions through a common latent factor—A population‐based twin study

**DOI:** 10.1002/jcv2.70069

**Published:** 2025-11-11

**Authors:** Torkel Carlsson, Henrik Larsson, Sebastian Lundström, Sven Bölte, Mark J. Taylor

**Affiliations:** ^1^ Center of Neurodevelopmental Disorders (KIND) Centre for Psychiatry Research Stockholm Sweden; ^2^ Department of Women's and Children's Health Karolinska Institutet & Stockholm Health Care Services Stockholm Sweden; ^3^ Child and Adolescent Psychiatry Stockholm Health Care Services Stockholm Sweden; ^4^ Department of Medical Epidemiology & Biostatistics Karolinska Institutet Stockholm Sweden; ^5^ Schools of Medical Sciences Örebro University Örebro Sweden; ^6^ Gillberg Neuropsychiatry Centre University of Gothenburg Gothenburg Sweden; ^7^ Centre for Ethics, Law, and Mental Health University of Gothenburg Gothenburg Sweden; ^8^ Curtin Autism Research Group Curtin School of Allied Health Curtin University Perth Western Australia

**Keywords:** cumulative environmental effect, early medical events, latent factor, neurodevelopmental conditions, twin study

## Abstract

**Background:**

The cumulative effect of early medical events has been shown to be associated with autism. It is unclear whether this effect is specific to autism or if it is associated to other neurodevelopmental conditions (NDCs) as well.

**Methods:**

We established a registry‐linked population‐based twin cohort of 10,254 pairs within the child and adolescent twin study in Sweden. Participants were evaluated for symptoms of autism, attention deficit hyperactivity disorder, tics, and learning difficulties at age 9. Three early medical events previously associated with autism, beyond familial confounding (low birth weight, congenital malformations, and perinatal respiratory stress), were summed up creating an individual cumulative exposure load. Confirmatory Factor Analysis was performed on the measure of NDCs and a standardized common latent NDC factor was regressed on level of cumulative exposure.

**Results:**

Cumulative exposure to early medical events was associated with a non‐linear increase in the common latent NDC factor, *ß* = 0.12 (95% CI, 0.07–0.17) for exposure level 1, *ß* = 0.25 (0.17–0.33) for exposure level 2, and *ß* = 0.62 (0.34–0.90) for exposure level 3. In a monozygotic twin difference analysis, with familial confounding being fully accounted for, the whole exposure effect was captured by the common factor, with residual associations fully attenuated for all four NDC outcomes, at all levels of cumulative exposure.

**Conclusion:**

The result suggests a possibly causal environmental pathway. The cumulative effect of early medical events previously associated with autism, are not specific for autism, but rather associated with NDCs in a broad sense.

## Key points

**What's known?**

- The cumulative effect of early medical events has been shown to be associated with autism. It is unclear whether this effect is specific to autism or associated to other neurodevelopmental conditions (NDCs) as well.

**What's new?**

- When studying environmental factors, it is important to account for familial confounding, since many suspected exposures and outcomes are in themselves heritable.
- In a registry‐linked population‐based twin cohort, with familial confounding being fully accounted, we found that cumulative exposure to early medical events was associated with an increase in a common latent NDC factor, suggesting a possibly causal environmental pathway.

**What's relevant?**

- Future research should study the etiology of NDCs by designs that separates genetic and environmental factors associated with the cumulative impact of early‐stage events.

## INTRODUCTION

Neurodevelopmental conditions (NDC) are characterized by alterations in the architecture and maturation of the brain causing challenges in cognitive functioning and impairments in social, educational, occupational, and other important areas of life (De Felice et al., 2015). NDCs are common, with a prevalence of 10%–15% (Aschner & Costa, 2015), comprising, among others, intellectual disability (ID), autism, attention deficit hyperactivity disorder (ADHD), and tic disorders (American Psychiatric Association, 2013). The causes of NDCs are multiple (Martin et al., 2018; Taylor et al., 2019) and the exact etiologies driving atypical neurodevelopment remain poorly understood. NDCs are in general highly heritable, with estimates ranging from 93% to 98% for ID, 64%–95% for autism, 77%–92% for ADHD and 70%–85% for tics disorders (Faraone & Larsson, 2019; Larsson et al., 2014; Lichtenstein et al., 2021; Mataix‐Cols et al., 2015; Polderman et al., 2015; Posthuma & Polderman, 2013; Ronald & Hoekstra, 2011; Tick et al., 2016). Nonetheless, heritability estimates leave space for significant environmental contributions (Herbert, 2010; Payne‐Sturges et al., 2021; Pessah et al., 2010; Shelton et al., 2012; Zuk et al., 2012).

When studying potential effects of environmental factors, it is important to account for familial confounding. Familial confounders include measured or unmeasured environmental and genetic factors, that are shared within a family, making family members similar. Since many suspected exposures and outcomes are in themselves to a degree heritable, it cannot be ruled out that associations are driven by familial links between the exposure and outcome, rather than being causal (D'Onofrio et al., 2013). Twin and sibling studies are well suited to adjust for familial confounding. A recent systematic review of twin and sibling studies on NDCs and early environmental exposures revealed advanced paternal age, low birth weight, malformations, and perinatal respiratory stress being associated with autism, and low birth weight, low gestational age and family income being associated with ADHD (Carlsson et al., 2021).

In addition to single factors, the cumulative stress hypothesis proposes that vulnerability for conditions, such as NDCs (Sarkar et al., 2019), is enhanced if adversities accumulate during early life (McEwen, 1998). Besides earlier evidence from animal studies (Nadeem et al., 2021; Pelch et al., 2019; Schaafsma et al., 2017) and smaller genetically informed studies (Chien et al., 2019; Isaksson et al., 2022; Willfors et al., 2017), we recently found that increasing cumulative exposure to early medical events (e.g., low birth weight, congenital malformations, perinatal respiratory stress, and advanced paternal age) was associated with increased likelihoods of autism and autistic symptoms (Carlsson et al., 2022).

Autism often co‐occurs with other NDCs, and there is evidence to suggest that this is due to shared genetic etiologies between autism and other NDCs (Ronald et al., 2008). A growing body of literature supports a transdiagnostic dimensional approach to NDCs that accounts for these shared underpinnings (Allegrini et al., 2019; Astle et al., 2022). It has been established a generally strong overlap between different NDCs, as they often co‐occur and may arise from overlapping genetic causes (Pettersson et al., 2013). It is, however, less clear whether environmental factors have the same general influence across NDCs, as *meta*‐anlaytic evidence demonstrates modest to moderate environmental influence (Michelini et al., 2024). It has, in particular, been shown that restricted fetal growth is associated with a small but significant increase in a latent general factor of psychopathology and a moderate increase in a specific latent NDC factor (Pettersson et al., 2019). Also, there is etiological—genetic and environmental—overlap between ADHD and autism symptoms (Ghirardi et al., 2019), as well as other NDCs (Lichtenstein et al., 2010), which is consistent with the notion of a general factor of NDCs (Pettersson et al., 2020). However, there is also independence within disorder constructs inconsistent with a general factor model (Happe & Ronald, 2008; Pettersson et al., 2013). For example, different symptoms of autism have been linked differentially to different symptoms of ADHD, with nonshared environmental correlations being lower than the genetic correlations (Taylor et al., 2015). Hence, it might be the case that while genetic factors operate on a general level across NDCs, environmental factors have more of a specific effect (Oerlemans et al., 2016). It is largely unknown whether this is the case, however, since one clear advantage with a transdiagnostic dimensional approach is the use of factor analysis, with its ability to discover and simplify underlying latent structures within a dataset of many interconnected variables, compared to, for example, multiple regression (Michelini et al., 2024; Oerlemans et al., 2016).

As far as we are aware, this is the first study with the aim to assess whether the association to cumulative exposure of early medical events is specific for autism, or if the association is more generally associated to other NDCs as well, including ADHD, tics and learning difficulties (LD), using a transdiagnostic dimensional approach.

We hypothesized that the environmental effect of cumulative early medical events previously associated with autism beyond familial confounding (Carlsson et al., 2021), is not specific for autism, but rather, that a cumulative effect is associated with a common latent NDC factor.

## SUBJECTS AND METHODS

### Participants

The study and the linkage of samples with registries was approved by the Regional Ethical Review Board in Stockholm County. The participants were twins taking part in the longitudinal, population‐based child and adolescent twin study in Sweden (CATSS), initiated in 2004 (Anckarsäter et al., 2011). CATSS involves inviting parents of 9‐year‐old twins born in Sweden to participate and complete a structured assessment of NDCs (earlier cohorts included 12‐year‐olds). With data collected from individuals born in every year between 1992 and 2008, the current study's cohort consisted of 10,254 monozygotic (MZ) and dizygotic (DZ) same sex twin pairs (Supporting Information S1: Table S1). Our measures show a large mean sex differences, and hence we excluded opposite‐sex twins to avoid inflating within‐pair differences in the analyses. CATSS, with a response rate of 75%, has been shown to have representative selected sample characteristics for the general population in Sweden (Taylor et al., 2020). Zygosity was determined by DNA analysis.

### Exposures

To study specificity of early adverse environmental factors for autism in relation to other NDCs, low birth weight, congenital malformations and perinatal hypoxia were examined, as they previously yielded associations with autism beyond familial confounding (Carlsson et al., 2021). We obtained detailed obstetric and neonatal information, as well as all diagnosis codes of interest for all participants throughout their lives, by linking CATSS to the Swedish medical birth register (Stephansson et al., 2018), and the national patient register (Ludvigsson et al., 2011), described in detail elsewhere (Carlsson et al., 2022). A binary variable was created to indicate whether each factor was present or not for each participant. An ordinal cumulative exposure load variable of early medical events was created by summing up the presence of binary factors for each participant. The sample distribution of the cumulative exposure ranged from 15,579 (76.0%) participants with a 0 score, to 141 (0.7%) with a score of 3 (Supporting Information S1: Table S1).

### Outcomes

In CATSS, all participants are evaluated for NDC symptoms at the age of 9 using the autism‐tics, ADHD and other comorbidities inventory (A‐TAC) (Hansson et al., 2005). Its validity is well established through clinical and population‐based samples, with excellent predictive properties for autism (area under the curve (AUC = 0.98), and ADHD (AUC = 0.93), and good for tics (AUC = 0.86) and LD (AUC = 0.87; Larson et al., 2013; Mårland et al., 2017). Items can be answered yes (scored as 1), yes, to a certain degree (0.5), or no (0). Seventeen items address autism, 19 address ADHD, three address tics, and three address LD, with skewed sample distributions of the scores (Supporting Information S1: Table S1).

### Statistics

This study used a within‐pair design to (a) examine the association between cumulative exposure to medical factors and NDCs (*Between all* analyses; i.e. do NDCs increase with increasing exposure to medical factors); and (b) test whether any observed associations are due to familial confounding (*Within twin* analyses; i.e. test whether any observed associations can be explained by genetic and shared environmental factors that influence both medical factors and NDCs).

#### Between all

First, confirmatory factor analysis (CFA) was performed using *R* version 4.1.1 and the lavaan package version 0.6–9 (Rosseel, 2012), in order to model a common latent NDC factor that captures variance common to the AUTISM, ADHD, tics and LD A‐TAC subscales. Standardized factor loadings for each A‐TAC subscale and fit statistics were calculated using maximum likelihood estimation with robust standard errors and a Satorra–Bentler scaled test statistic. Scaled and robust root mean square error of approximation (RMSEA) were used to evaluate model fit, with RMSEA at 0.05 considered good. Second, the effect of the standardized common latent NDC factor was regressed out from each A‐TAC subscale, respectively, using linear regressions, creating residual outcome variance scores for each individual. Third, a linear regression of the common latent NDC factor on level of cumulative exposure was performed, both crude and adjusted for sex and birthyear, with robust Sandwich estimators to account for related individuals in the sample, using the *R* package drgee (Zetterqvist & Sjölander, 2015). Fourth, linear regressions of the respective outcome residuals on level of cumulative exposure, were performed to test the degree to which cumulative exposure was associated with each NDC after adjusting for the common latent NDC factor.

#### Within twin

Using the twin difference design (Lim et al., 2018) to control for genetic and shared environmental influences, while modeling a common latent NDC factor, we calculated within twin pair difference scores for each twin pair, both for the cumulative exposure and for each A‐TAC subscale (Supporting Information S1: Appendix S1, Table S2, Figure S1). Standardized factor loadings and fit statistics were calculated, and the effect of the common latent NDC factor were regressed out from each outcome, as above. Then a linear regression of the standardized common latent NDC factor on level of cumulative exposure twin difference was performed. To test the degree to which within twin pair cumulative exposure difference was associated with each A‐TAC subscale difference after adjusting for the common latent NDC factor, linear regressions were performed on the respective residual outcome difference variances on level of cumulative exposure difference. Finally, to fully account for familial confounding the same approach was used for a within twin analysis grouped by zygosity, since MZ twins share almost 100% of their genetics while DZ twins only share 50%. A common latent NDC factor was introduced as before, to create a within twin pair split by zygosity model, with the above steps reperformed on MZ and DZ twin pairs separately. As the within‐pair comparisons were less well powered than the between individual comparisons, we truncated the highest levels of exposure (2 and 3 exposures) into a single category (2 or more exposures).

## RESULTS

We used CFA to fit the three correlated factor models to the four A‐TAC subscale outcomes, and all models fit the data well (Supporting Information S1: Appendix S1, Figures S2–S4, Tables S3–S4).

### Linear regressions of level of cumulative early medical events—Between all participants

We regressed the standardized common latent NDC factor on level of cumulative exposure, with the estimates from the crude regressions being almost identical to the sex and birthyear adjusted ones (Table 1). There was an increase of the NDC factor by level of exposure, with *ß* = 0.12 (95% CI, 0.07 to 0.17) for exposure level 1, *ß* = 0.25 (95% CI, 0.17 to 0.33) for exposure level 2, and *ß* = 0.62 (95% CI, 0.34 to 0.90) for exposure level 3. Last, we looked for exposure effects on the respective outcomes not captured by the common latent NDC factor, by regressing the residual outcome variances on level of cumulative exposure. These effects were all small or negative at all exposure levels ranging from *ß* = 0.00 (95% CI, −0.02 to 0.02) to *ß* = −0.09 (95% CI, −0.24 to 0.06) for autism, *ß* = −0.01 (95% CI, −0.06 to 0.05) to *ß* = −0.16 (95% CI, −0.49 to 0.17) for ADHD, and *ß* = −0.02 (95% CI, −0.03 to 0.00) for tics, with slightly larger effects found for residual LD and the exposure levels 2 (*ß* = 0.10 [95% CI, 0.07 to 0.13]) and 3 (*ß* = 0.26 [95% CI, 0.13 to 0.38]) (Table 1).

**TABLE 1:** Linear regressions of common latent NDC factor and residual outcome variances on level of cumulative exposure.

Mode/sample		Regression	Exposure level 1				Exposure level 2				Exposure level 3			
			*ß*	(95% CI)	SE	*p*	*ß*	(95% CI)	SE	*p*	ß	(95% CI)	SE	*p*
**Between all**	Crude	Exp ∼ NDC	**0.14**	**(0.09–0.19)**	**0.03**	**<.001**	**0.28**	**(0.20–0.36)**	**0.04**	**<.001**	**0.63**	**(0.35–0.91)**	**0.14**	**<.001**
		Exp ∼ resid. Autism	*0.00*	*(−0.03–0.02)*	*0.01*	*.876*	***−0.06***	***(−0.10–−0.01)***	*0.02*	*.008*	*−0.09*	*(−0.24–0.05)*	*0.08*	*.216*
		Exp ∼ resid. ADHD	*0.00*	*(−0.06–0.05)*	*0.03*	*.927*	*0.01*	*(−0.08–0.10)*	*0.05*	*.798*	*−0.17*	*(−0.51–0.16)*	*0.17*	*.302*
		Exp ∼ resid. Tics	*−0.02*	*(−0.03–0.00)*	*0.01*	*.040*	***−0.03***	***(−0.06–−0.01)***	*0.01*	*.010*	*−0.06*	*(−0.15–0.02)*	*0.04*	*.160*
		Exp ∼ resid. LD	*0.02*	*(0.00–0.04)*	*0.01*	*.068*	***0.09***	***(0.06–0.12)***	*0.02*	*<.001*	***0.27***	***(0.14–0.39)***	*0.06*	*<.001*
	Adjusted	Exp ∼ NDC^a^	**0.12**	**(0.07–0.17)**	**0.03**	**<.001**	**0.25**	**(0.17–0.33)**	**0.04**	**<.001**	**0.62**	**(0.34–0.90)**	**0.14**	**<.001**
		Exp ∼ resid. Autism	*0.00*	*(−0.02–0.02)*	*0.01*	*.982*	***−0.06***	***(−0.10–−0.01)***	*0.02*	*.010*	*−0.09*	*(−0.24–0.06)*	*0.08*	*.228*
		Exp ∼ resid. ADHD	*−0.01*	*(−0.06–0.05)*	*0.03*	*.810*	*0.00*	*(−0.09–0.09)*	*0.05*	*.996*	*−0.16*	*(−0.49–0.17)*	*0.17*	*.330*
		Exp ∼ resid. Tics	*−0.02*	*(−0.03–0.00)*	*0.01*	*.024*	***−0.03***	***(−0.06–−0.01)***	*0.01*	*.007*	*−0.06*	*(−0.15–0.02)*	*0.04*	*.152*
		Exp ∼ resid. LD	*0.02*	*(0.00–0.04)*	*0.01*	*.046*	***0.10***	***(0.07–0.13)***	*0.02*	*<.001*	***0.26***	***(0.13–0.38)***	*0.06*	*<.001*

Mode/sample		Regression	Exposure difference level = 1				Exposure difference level ≥ 2			
			*ß*	(95% CI)	SE	*p*	*ß*	(95% CI)	SE	*p*
**Within twins**	Whole sample	Exp ∼ NDC	**0.10**	**(0.05–0.16)**	**0.03**	**<.001**	**0.25**	**(0.05–0.45)**	**0.10**	**.016**
		Exp ∼ resid. Autism	*0.00*	*(−0.04–0.04)*	*0.02*	*.950*	*0.03*	*(−0.04–0.04)*	*0.07*	*.652*
		Exp ∼ resid. ADHD	*0.03*	*(−0.07–0.14)*	*0.05*	*.519*	*−0.15*	*(−0.53–0.23)*	*0.20*	*.443*
		Exp ∼ resid. Tics	*0.00*	*(−0.03–0.02)*	*0.01*	*.932*	***−0.12***	***(−0.21–−0.03)***	*0.05*	*.013*
		Exp ∼ resid. LD	*−0.01*	*(−0.04–0.02)*	*0.01*	*.466*	*0.10*	*(0.00–0.21)*	*0.05*	*.043*
	MZ	Exp ∼ NDC	**0.08**	**(0.03–0.13)**	**0.03**	**.003**	**0.25**	**(0.10–0.41)**	**0.08**	**.001**
		Exp ∼ resid. Autism	*0.04*	*(−0.02–0.09)*	*0.03*	*.191*	*0.00*	*(−0.16–0.16)*	*0.08*	*.987*
		Exp ∼ resid. ADHD	*−0.08*	*(−0.19–0.02)*	*0.05*	*.109*	*0.01*	*(−0.30–0.32)*	*0.16*	*.941*
		Exp ∼ resid. Tics	*0.02*	*(−0.01–0.06)*	*0.02*	*.191*	*−0.04*	*(−0.15–0.06)*	*0.05*	*.411*
		Exp ∼ resid. LD	*0.00*	*(−0.03–0.03)*	*0.01*	*.829*	*0.01*	*(−0.07–0.10)*	*0.05*	*.744*
	DZ	Exp ∼ NDC	**0.11**	**(0.02–0.21)**	**0.05**	**.020**	0.20	(−0.22 to 0.63)	**0.22**	**.344**
		Exp ∼ resid. Autism	*−0.02*	*(−0.08–0.04)*	*0.03*	*.549*	*0.14*	*(−0.12–0.40)*	*0.13*	*.298*
		Exp ∼ resid. ADHD	*0.14*	*(−0.03–0.32)*	*0.09*	*.111*	*−0.63*	*(−1.41–0.15)*	*0.40*	*.115*
		Exp ∼ resid. Tics	*−0.02*	*(−0.06–0.02)*	*0.02*	*.346*	***−0.23***	***(−0.41–−0.06)***	*0.09*	*.008*
		Exp ∼ resid. LD	*−0.01*	*(−0.06–0.03)*	*0.02*	*.575*	***0.26***	***(0.06–0.47)***	*0.10*	*.011*

*Note:* Residual variance regressions in italic. Statistically significant results in bold.

Abbreviations: ADHD, attention deficit hyperactivity disorder symptoms; Autism, autism symptoms; DZ, dizygotic; Exp ∼ resid, regression of exposure level and residual variance; LD, learning difficulties; MZ, monozygotic; NDC, neurodevelopmental condition latent factor.

^a^ Adjusted for sex (*ß* = 0.73, SE = 0.03, *p* =< .001) and birthyear (*ß* = 1.02, SE = 0.00, *p* =< .001).

### Within twin pairs

To account for familial confounding, we regressed the standardized common latent NDC factor on level of cumulative exposure difference within twin pairs (Table 1). Supporting Information S1: Table S2 shows the number of informative pairs for these analyses, showing the numbers who were discordant for the outcome, exposure, and both. The associations of the common latent NDC factor on level of cumulative exposure difference in the whole sample existed beyond familial confounding, with *ß* = 0.10 (95% CI, 0.05 to 0.16) for exposure difference level 1, and *ß* = 0.25 (95% CI, 0.05 to 0.45) for exposure difference level ≥ 2. The residual outcome effects, not captured by the common latent NDC factor, were all small or negative at all exposure difference levels with *ß* = 0.00 (95% CI, −0.04 to 0.04) to *ß* = 0.03 (95% CI, −0.04 to 0.04) for autism, *ß* = −0.03 (95% CI, −0.07 to 0.14) to *ß* = −0.15 (95% CI, −0.53 to 0.23) for ADHD, *ß* = 0.00 (95% CI, −0.03 to 0.02) to *ß* = −0.12 (95% CI, −0.21 to 0.03) for tics, and *ß* = −0.01 (95% CI, −0.04 to 0.02) to *ß* = 0.10 (95% CI, 0.00 to 0.21) for LD (Table 1).

### Split by zygosity

There was an association between cumulative exposure to medical factors and the latent NDC factor for the MZ subsample with *ß* = 0.08 (95% CI, 0.03 to 0.13) for exposure level difference 1, and *ß* = 0.25 (95% CI, 0.10 to 0.41) for exposure level difference ≥ 2, and for the DZ subsample with *ß* = 0.11 (95% CI, 0.02 to 0.21) for exposure level difference 1, and for exposure level difference ≥ 2 (*ß* = 0.20 (95% CI, −0.22 to 0.63), albeit with confidence intervals that overlapped with 0. The residual outcome effects, not captured by the common latent NDC factor, were similar, but in some instances slightly larger, in the DZ subsample compared to the whole sample. When familial confounding was fully accounted for, as in the MZ‐subsample, the residual associations for all outcomes fully attenuated, at all levels of cumulative exposure difference (Table 1).

## DISCUSSION

In this population‐based registry‐linked twin study, the cumulative effect of early medical events that previously have been shown to be associated with autism beyond familial confounding, were similarly associated with symptoms of ADHD, tics and LD, through a common latent NDC factor. The existence of an association beyond familial confounding supports a causal association between cumulative exposure to early medical events not only in autism, but NDCs more generally. As familial confounding was fully accounted for in a large population‐based twin sample, with full attenuation of residual outcome associations in the MZ‐subsample, albeit this result should be interpreted considering the lower power of the MZ subsample analyses.

We first want to consider these results in light of concerns by the autistic community about medical research. This study poses a medical research question (i.e., testing whether early medical exposures are linked with NDCs), yet it has been proposed that autism research should adopt a social model of disability (Pellicano & den Houting, 2022). We would first highlight that we report generally small associations between early medical exposures and later NDCs here. The results should not be taken to mean that such factors can be used to predict or prevent autism and other NDCs. Second, autistic individuals report high rates of co‐occurring somatic conditions across their lifetimes (Williams & Gotham, 2022). Studies such as ours can draw attention to potential mechanisms underlying these associations, such as whether genetic factors play a role. This can inform future study designs, for example, should such studies *adjust* for genetic factors.

There was an increase in the strength of association to NDC by level of exposure, also adding support to the hypothesis of an additive effect, in line with earlier findings on tic disorder (Brander et al., 2017). The accumulation of exposure effect, points to a background mechanism in accordance with the cumulative stress hypothesis (Sarkar et al., 2019). Possible underlying mechanisms within the cumulative stress hypothesis involves for example, environmental pollutants (Roberts et al., 2013), endocrine disruptors (Cheroni et al., 2020), or stress hormones (McEwen, 1998), underpinning alterations in the architecture and maturation of the brain (De Felice et al., 2015). Our genetically informed study adds support to the proposed anterior modifiers in the emergence of neurodevelopmental disorders framework, which advise future research to elucidate the etiology of NDCs by prospective longitudinal designs that separates genetic and environmental factors associated with the cumulative impact of early‐stage events (Johnson et al., 2021).

The finding of ADHD and autism being similarly impacted is particularly intriguing. In the growing body of literature on the framework of a general factor of neurodevelopmental disorders (Lichtenstein et al., 2010), earlier findings have highlighted the role of genetics, with environmental contributions being less clear. It has been shown that restricted fetal growth is associated with a moderate increase in a latent NDC factor (Pettersson et al., 2019). But, different symptoms of autism have also been linked differentially to different symptoms of ADHD, with nonshared environmental correlations being lower than the genetic correlations (Taylor et al., 2019), and research has indicated that pre‐ and perinatal risk factors might play a role in diverging developmental pathways leading up to either disorder (Oerlemans et al., 2016). What explains this difference in results between these studies and ours might be our cumulative approach to the exposures studied. It should also be noted that cumulative exposure to early medical events was associated with LD independently of the general factor, further indicating that environmental factors could partly differentiate these NDCs from other NDCs.

For our hypothesis driven study, we only choose the environmental factors that in a recent comprehensive systematic review had been found to be associated with autism beyond familial confounding (Carlsson et al., 2021). Since these environmental factors only were early medical factors, we were able to measure them by linking national medical registries to our sample. This might be an example of the streetlight effect (Battaglia & Atkinson, 2015)—our list of factors originating from research studying only what has been possible to study. Furthermore, our included factors all stem from its known association to autism in particular, and not to NDCs in general, making our list of factors even more selective. Still, and perhaps further convincing, despite not including other pertinent environmental exposures, our result shows the importance of the accumulation of environmental factors in the etiology of NDCs.

### Strengths and limitations

Our study has several strengths. First, our large population‐based twin sample enables the investigation of even weak, yet important, environmental contributions, while adjusting for familial confounding. Second, by linking our cohort to medical registries we retrieved prospectively collected data of good quality. Third, by comparing twins we ruled out the effect of parental genetics influencing our result, and by comparing the within pair difference of MZ twins we also accounted for the influence of child genetics.

Limitations need to be addressed. First, the cumulative score was created by summing exposures based on them being present or absent, and therefore, we were not able to account for the possibility of different effect sizes for each exposure. Second, as for all observational studies the risk of residual bias prevents far reaching causal inferences. Third, despite our large sample, the skewed distributions of the data probably complicated the model fitting leading to higher robust RMSEA. However, fit indices are to be interpreted holistically (Hayduk et al., 2007). Fourth, generalizability from twins to singletons needs consideration, especially since we saw a slightly higher share of MZ pairs having a higher exposure load (Table 1). However, the literature on the role of zygosity for perinatal outcomes is scarce. Zygosity has in a previous retrospective study been associated to lower birth weight and more prematurity (Hoskins, 1995), while in a later study the effect of zygosity was less clear (Dube et al., 2002). Also, in a prior study using the same cumulative exposure, the results did not differ when twin pairs with twin‐to‐twin transfusion syndrome were excluded (Carlsson et al., 2022). Fifth, measurement error at the within‐pair level needs to be addressed, although our exposures have been extensively studied before (Carlsson et al., 2022). The presence of a measurement error or misclassification of a continuous variable leads to an attenuation of both the between individuals and the within pair association, compared to the true association, with within‐pair associations being more attenuated than between‐pair associations (Frisell et al., 2012). Finally, while we believe it is a strength of the study that we selected early medical exposures based on previous literature, we only included three exposures. Other exposures are likely to be relevant to NDCs, such as advanced paternal age (D’Onofrio et al., 2014), are likely to be pertinent and were not included here. Indeed, as methodology to more systematically identify environmental factors (such as “exposome‐wide association studies”) develop, we expect future studies will need to focus on a larger array of exposures when examining cumulative exposure to environmental factors.

A final point to note is that the coverage of NDCs was relatively limited here, with this study focusing on four NDCs. However, NDCs are a broad category within DSM‐5, which also incorporates developmental language disorder and ID. These conditions were not measured here. Indeed, given the lack of evidence on the etiology of these conditions (Carlsson et al., 2022), they should be incorporated in future studies of environmental influences on NDCs.

## CONCLUSION

This study connects the exposure of cumulative early medical events to NDCs through a common latent factor, beyond familial confounding, suggesting a causal pathway. Our genetically informed study confirms the hypothesis that the environmental effect of cumulative early medical events previously associated with autism beyond familial confounding, is not specific for autism, but rather, associated with a common latent NDC factor that in turn affects symptoms of NDC, autism included.

## AUTHOR CONTRIBUTIONS

**Torkel Carlsson**: Conceptualization; data curation; formal analysis; funding acquisition; investigation; methodology; project administration; software; visualization; writing—original draft. **Henrik Larsson**: Validation; writing—review and editing. **Sebastian Lundström**: Funding acquisition; resources; validation; writing—review and editing. **Sven Bölte**: Conceptualization; funding acquisition; supervision; validation; writing—review and editing. **Mark J. Taylor**: Conceptualization; formal analysis; methodology; software; supervision; validation; writing—review and editing.

## CONFLICT OF INTEREST STATEMENT

S.B. discloses that he has in the last 4 years acted as an author, consultant or lecturer for Medice and Roche. He receives royalties for textbooks and diagnostic tools from Hogrefe. H.L. reports receiving grants from Shire Pharmaceuticals; personal fees from and serving as a speaker for Medice, Shire/Takeda Pharmaceuticals and Evolan Pharma AB; and sponsorship for a conference on attention‐deficit/hyperactivity disorder from Shire/Takeda Pharmaceuticals and Evolan Pharma AB. The remaining authors have declared that they have no competing or potential conflicts of interest.

## ETHICAL CONSIDERATIONS

Participation in the study is based on informed consent. The study and the linkage of samples with registries was approved by the Regional Ethical Review Board in Stockholm County: DNR2016/2135‐31 the August 12, 2018; DNR 2018/2013‐32 the December 21, 2017; and DNR 2020–04248 the August 21, 2020.

## Supporting information

Supporting Information S1

## Data Availability

The Public Access to Information and Secrecy Act in Sweden prohibits the authors from making individual level data publicly available. Researchers who are interested in replicating the work can apply for individual level data at the Swedish Twin Registry (https://ki.se/en/research/swedish‐twin‐registry‐for‐researchers).
