# Supplementary material for: Association of cumulative early medical events and neurodevelopmental conditions through a common latent factor—A population‐based twin study
Source: JCPP Adv. 2025 Nov 11;6(3):e70069. doi: 10.1002/jcv2.70069 (PMC13339276; doi:10.1002/jcv2.70069)
Supplement: Supplementary file 1 [file JCV2-6-e70069-s001.docx]

**Association of Cumulative Early Medical Events and Neurodevelopmental Conditions Through a Common Latent Factor – a Population-Based Twin Study**

Supporting information

**Table of content**

Table S1. Sample characteristics	2

Appendix S1. Information on simultaneously exposure and outcome discordant pairs	3

Figure S1. Histograms depicting the distribution of outcome discordancy within twin pairs in

relation to simultaneous exposure discordancy	3

Table S2. Outcome discordant pairs with simultaneous exposure discordancy	4

Table S3. Model fit information and standardized factor loadings for the common latent NDC

factor models	5

Figure S2. The Common Latent NDC Factor Model – Between All Participants, with

standardized factor loadings	6

Figure S3. The Common Latent NDC Factor Model – Within Twin Pairs, with standardized

factor loadings	6

Figure S4. The Common Latent NDC Factor Model – Within Twin Pairs Split by Zygosity,

with standardized factor loadings	7

Table S4. Outcome Correlation Matrices	7

**Table S 1. Sample characteristics**

A-TAC scores

| Total *N* individuals (%) | Whole sample | No exposure | Exposure level = 1 | Exposure level = 2 | Exposure level = 3 |
| --- | --- | --- | --- | --- | --- |
|  | 20508 | 15579 (76.0) | 3448 (16.8) | 1340 (6.5) | 141 (0.7) |
| ASD, mean (SD) | 0.86 (1.65) | 0.81 (1.58) | 0.97 (1.81) | 1.10 (1.98) | 1.46 (2.28) |
| ADHD, mean (SD) | 2.13 (3.19) | 2.03 (3.07) | 2.34 (3.42) | 2.67 (3.65) | 3.26 (4.00) |
| Tics, mean (SD) | 0.17 (0.47) | 0.17 (0.46) | 0.18 (0.49) | 0.19 (0.50) | 0.22 (0.57) |
| Learning difficulties, mean (SD) | 0.29 (0.62) | 0.26 (0.58) | 0.32 (0.67) | 0.44 (0.76) | 0.71 (0.99) |
| Median birthyear (IQR) | 2000 (1996, 2004) | 2000 (1996, 2004) | 2000 (1996, 2004) | 2001 (1996, 2005) | 1997 (1994, 2004) |
| *N* of females (%) | 10026 (48.9) | 7809 (50.1) | 1554 (45.1) | 602 (44.9) | 61 (43.3) |
| *N* of MZ twins (%) | 9446 (46.1) | 6943 (44.6) | 1681 (48.8) | 742 (55.4) | 80 (56.7) |
| *N* of DZ twins (%) | 11062 (53.9) | 8636 (55.4) | 1767 (51.2) | 598 (44.6) | 61 (43.3) |

*A-TAC* the Autism–Tics, ADHD and Other Comorbidities Inventory, *MZ* monozygotic, *DZ* dizygotic

**Appendix S 1 . Information on simultaneously exposure and outcome discordant pairs**

The outcome discordant pairs are either simultaneously exposure discordant or not exposure discordant. Furthermore, the simultaneous outcome and exposure discordancy is either in the direction that the twin with more symptoms has more exposures than their co-twin, or reversed, that the twin with more symptoms has less exposures. In addition, the level of outcome and exposure discordancy is of interest in the within twin analysis, with the histograms depicting the distribution of outcome discordancy in relation to exposure discordance for each outcome shown in Figure S1.

**Figure S 1. Histograms depicting the distribution of outcome discordancy within twin pairs in relation to simultaneous exposure discordancy**

5000

4000

3000

2000

Number of pairs

1000

ASD

3000

2000

Number of pairs

1000

ADHD

Doubly discordant

0	5	10	15

0	5	10	15

Yes No

^20^	Reversed

8000

6000

Tics

Intrapair ASD symptom difference

6000

Intrapair ADHD symptom difference

Learning Difficulties

doubleDisAsd

−1

0

1

4000

Number of pairs

2000

0	1	2	3

Intrapair Tics symptom difference

4000

2000

Number of pairs

0	1	2	3

Intrapair Learning Difficulties symptom difference

For each twin pair a within twin difference score was calculated for the cumulative exposure and for each outcome, respectively. The outcome discordant pairs in our sample are either simultaneously exposure discordant (doubly discordant) or not exposure discordant. The simultaneous outcome and exposure discordancy is either in the direction that the twin with more symptoms has more exposure than their co-twin, or reversed, that the twin with more symptoms has less exposure.

**Table S 2. Outcome discordant pairs with simultaneous exposure discordancy**

|  | **Exposure  discordant** |  |  |  | **Exposure  discordancy** |  |
| --- | --- | --- | --- | --- | --- | --- |
|  | Type | N (%) | **Level of ourcome  discordancy, mean (SD)** | *From 0 to 1* | *From 0 to 2* | *From 1 to 2* |
| Entire Sample |  |  |  |  |  |  |
| *Autism Discordant* | Yes | 539 (27.3%) | 1.50 (1.71) | 412 (27.3%) | 21 (20.4%) | 106 (29.4%) |
|  | No | 798 (40.5%) | 0.74 (1.45) | 622 (41.2%) | 42 (40.8%) | 134 (37.2%) |
|  | Reversed | 634 (32.2%) | 1.79 (2.10) | 474 (31.4%) | 40 (38.8%) | 120 (33.3%) |
| *ADHD Discordant* | Yes | 701 (35.6%) | 2.81 (2.90) | 534 (35.4%) | 33 (32.0%) | 134 (37.2%) |
|  | No | 543 (27.5%) | 1.86 (2.73) | 428 (28.4%) | 30 (29.1%) | 85 (23.6%) |
|  | Reversed | 727 (36.9%) | 3.32 (3.24) | 546 (36.2%) | 40 (38.8%) | 141 (39.2%) |
| *Tics Disorcdant* | Yes | 254 (12.9%) | 0.94 (0.61) | 198 (13.1%) | 18 (17.5%) | 38 (10.6%) |
|  | No | 1447 (73.4%) | 0.20 (0.49) | 1103 (73.1%) | 75 (72.8%) | 269 (74.7%) |
|  | Reversed | 270 (13.7%) | 0.99 (0.64) | 207 (13.7%) | 10 (9.7%) | 53 (14.7%) |
| *Learning Discordant* | Yes | 338 (17.1%) | 1.07 (0.71) | 264 (17.5%) | 10 (9.7%) | 64 (17.8%) |
|  | No | 1262 (64.0%) | 0.28 (0.57) | 975 (64.7%) | 70 (68.0%) | 217 (60.3%) |
|  | Reversed | 371 (18.8%) | 1.16 (0.76) | 269 (17.8%) | 23 (22.3%) | 79 (21.9%) |
| MZ |  |  |  |  |  |  |
| *Autism Discordant* | Yes | 222 (24.2%) | 1.25 (1.36) | 154 (23.2%) | 11 (17.2%) | 57 (29.7%) |
|  | No | 434 (47.2%) | 0.50 (0.97) | 336 (50.7%) | 30 (46.9%) | 68 (35.4%) |
|  | Reversed | 263 (28.6%) | 1.57 (1.89) | 173 (26.1%) | 23 (35.9%) | 67 (34.9%) |
| *ADHD Discordant* | Yes | 293 (31.9%) | 2.26 (2.24) | 204 (30.8%) | 17 (26.6%) | 72 (37.5%) |
|  | No | 314 (34.2%) | 1.25 (1.97) | 247 (37.3%) | 24 (37.5%) | 150 (78.1%) |
|  | Reversed | 312 (33.9%) | 2.63 (2.70) | 212 (32.0%) | 23 (35.9%) | 77 (40.1%) |
| *Tics Discordant* | Yes | 102 (11.1%) | 0.90 (0.55) | 77 (11.6%) | 9 (14.1%) | 16 (8.3%) |
|  | No | 708 (77.0%) | 0.15 (0.40) | 510 (76.9%) | 48 (75.0%) | 150 (78.1%) |
|  | Reversed | 109 (11.9%) | 1.05 (0.67) | 76 (11.5%) | 7 (10.9%) | 26 (13.5%) |
| *Learning Discordant* | Yes | 108 (11.8%) | 1.00 (0.66) | 79 (11.9%) | 5 (7.8%) | 24 (12.5%) |
|  | No | 672 (73.1%) | 0.16 (0.39) | 497 (75.0%) | 49 (76.6%) | 126 (65.6%) |
|  | Reversed | 139 (15.1%) | 1.01 (0.65) | 87 (13.1%) | 10 (15.6%) | 42 (21.9%) |
| DZ |  |  |  |  |  |  |
| *Autism Discordant* | Yes | 317 (30.1%) | 1.67 (1.91) | 258 (30.5%) | 10 (25.6%) | 49 (29.2%) |
|  | No | 364 (34.6%) | 0.95 (1.75) | 286 (33.8%) | 12 (30.8%) | 66 (39.3%) |
|  | Reversed | 371 (35.3%) | 1.94 (2.23) | 301 (35.6%) | 17 (43.6%) | 53 (31.5%) |
| *ADHD Discordant* | Yes | 408 (38.8%) | 3.21 (3.24) | 330 (39.1%) | 16 (41.0%) | 62 (36.9%) |
|  | No | 229 (21.8%) | 2.40 (3.15) | 171 (21.4%) | 6 (15.4%) | 42 (25.0%) |
|  | Reversed | 415 (39.4%) | 3.83 (3.50) | 334 (39.5%) | 17 (43.6%) | 64 (38.1%) |
| *Tics Discordant* | Yes | 152 (14.4%) | 0.97 (0.64) | 121 (14.3%) | 9 (23.1%) | 22 (13.1%) |
|  | No | 739 (70.2%) | 0.25 (0.55) | 593 (70.2%) | 27 (69.2%) | 110 (70.8%) |
|  | Reversed | 161 (15.3%) | 0.96 (0.62) | 131 (15.5%) | <5 | 27 (16.1%) |
| *Learning Discordant* | Yes | 230 (21.9%) | 1.10 (0.73) | 185 (21.9%) | 5 (12.8%) | 40 (23.8%) |
|  | No | 590 (56.1%) | 0.38 (0.67) | 478 (56.6%) | 21 (53.8%) | 91 (54.2%) |
|  | Reversed | 232 (22.1%) | 1.26 (0.81) | 182 (21.5%) | 13 (33.3%) | 37 (22.0%) |

*The table shows the number of individuals who were discordant for the exposures and outcomes. ‘Yes’ refers to pairs where the twin pairs were discordant in the same direction; that is, one twin had both a higher cumulative load and a higher outcome score. ‘Reversed’ refers to pairs that were discordant in different directions; that is, one twin had a higher cumulative load but lower outcome score (or vice versa). ‘No’ means that the pairs were not exposure discordant.*

**Table S 3. Model fit information and standardized factor loadings for the common latent NDC factor models**

| **Model** | **Sample** | **Outcome** | **ß** | **(95%CI)^1^** | **SE** | **Par.** | **df** | **RMSEA** | ***p* RMSEA** | **rob. RMSEA** | **(90%CI)** |
| --- | --- | --- | --- | --- | --- | --- | --- | --- | --- | --- | --- |
|  |  | ASD | 0.84 | (0.82–0.85) | 0.01 |  |  |  |  |  |  |
| Between all | Whole sample | ADHD  Tics | 0.81  0.43 | (0.79–0.82)  (0.4–0.45) | 0.01  0.01 | 8 | 2 | 0.058 | 0.004 | 0.093 | (0.080-0.106) |
|  |  | Learning difficulties | 0.56 | (0.54–0.58) | 0.01 |  |  |  |  |  |  |
|  |  | ASD | 0.75 | (0.72–0.79) | 0.02 |  |  |  |  |  |  |
| Within pairs | Whole sample | ADHD  Tics | 0.67  0.35 | (0.64–0.7)  (0.32–0.39) | 0.01  0.02 | 8 | 2 | 0.053 | 0.23 | 0.080 | (0.064-0.098) |
|  |  | Learning difficulties | 0.51 | (0.48–0.54) | 0.02 |  |  |  |  |  |  |
|  |  | ASD | 0.78 | (0.75–0.82) | 0.02 |  |  |  |  |  |  |
|  | MZ | ADHD  Tics | 0.67  0.39 | (0.63–0.7)  (0.35–0.44) | 0.02  0.02 |  |  |  |  |  |  |
| Within pairs,  grouped by zygosity |  | Learning difficulties | 0.51 | (0.48–0.55) | 0.02 | 24 | 4 | 0.051 | 0.392 | 0.075 | (0.058-0.093 |
|  |  | ASD | 0.67 | (0.61–0.73) | 0.03 |  |  |  |  |  |  |
|  | DZ | ADHD  Tics | 0.68  0.25 | (0.63–0.74)  (0.19–0.31) | 0.03  0.03 |  |  |  |  |  |  |
|  |  | Learning difficulties | 0.48 | (0.43–0.54) | 0.03 |  |  |  |  |  |  |

1 All p-values <0.001

*ß=standardized path coefficients from the latent common factor to each specific measure, including 95% confidence intervals and standard errors (SE). Par.: number of paramters in the model; df: degrees of freedom; RMSEA: root mean square error of approximation; p RMSEA: p-values for RMSEA; rob. RMSEA: robust estimate of RMSEA, accounting for non-independent observations in the data; 90% CI: 90% confidence intervals around the robust RMSEA estimate.*

Carlsson et al. 2022	Association of Cumulative Early Medical Events and Common Latent NDC Factor	6

**Figure S 2. The Common Latent NDC Factor Model – Between All Participants, with standardized factor loadings**

Confirmatory Factor Analysis (CFA) introducing a common latent NDC-factor to create a Common Latent NDC Factor Model – Between All Participants. Standardized factor loadings (95%CI) for each A-TAC subscale.

***= p<0.001, *Learn. Diffic.* Learning difficulties

**Figure S 3. The Common Latent NDC Factor Model – Within Twin Pairs, with standardized factor loadings**

Confirmatory Factor Analysis (CFA) introducing a common latent NDC-factor to create a Common Latent NDC Factor Model – Within Twin Pairs. Standardized factor loadings (95%CI) for each A-TAC subscale.

***= p<0.001, *Learn. Diffic.* Learning difficulties

Carlsson et al. 2022	Association of Cumulative Early Medical Events and Common Latent NDC Factor	7

**Figure S 4. The Common Latent NDC Factor Model – Within Twin Pairs Split by Zygosity, with standardized factor loadings**

Confirmatory Factor Analysis (CFA) introducing a common latent NDC-factor to create a Common Latent NDC Factor Model – Within Twin Pairs Split by Zygosity. Standardized factor loadings (95%CI) for each A-TAC subscale.

***= p<0.001, *MZ* Monozygotic, *DZ* Dizygotic, *Learn. Diffic.* Learning difficulties

**Table S 4 . Outcome Correlation Matrices**

| **Between all** | | | | |
| --- | --- | --- | --- | --- |
|  | ASD | ADHD | Tics | LD |
| ASD | 1 | 0,67 | 0,38 | 0,47 |
| ADHD | 0,67 | 1 | 0,34 | 0,48 |
| Tics | 0,38 | 0,34 | 1 | 0,16 |
| LD | 0,47 | 0,48 | 0,16 | 1 |

| **Within twin** | | | | |
| --- | --- | --- | --- | --- |
|  | ASD | ADHD | Tics | LD |
| ASD | 1 | 0,50 | 0,30 | 0,39 |
| ADHD | 0,50 | 1 | 0,23 | 0,37 |
| Tics | 0,30 | 0,23 | 1 | 0,11 |
| LD | 0,39 | 0,37 | 0,11 | 1 |

*LD* Learning difficulties
